# Multiple Myeloma and Kidney Disease

**DOI:** 10.1155/2013/487285

**Published:** 2013-10-27

**Authors:** Daisuke Katagiri, Eisei Noiri, Fumihiko Hinoshita

**Affiliations:** ^1^Department of Nephrology and Endocrinology, University Hospital, University of Tokyo, 7-3-1 Hongo, Bunkyo, Tokyo 113-8655, Japan; ^2^Department of Nephrology, National Center for Global Health and Medicine, 1-21-1 Toyama, Shinjyuku, Tokyo 162-8655, Japan

## Abstract

Multiple myeloma (MM) has a high incidence rate in the elderly. Responsiveness to treatments differs considerably among patients because of high heterogeneity of MM. Chronic kidney disease (CKD) is a common clinical feature in MM patients, and treatment-related mortality and morbidity are higher in MM patients with CKD than in patients with normal renal function. Recent advances in diagnostic tests, chemotherapy agents, and dialysis techniques are providing clinicians with novel approaches for the management of MM patients with CKD. Once reversible factors, such as hypercalcemia, have been corrected, the most common cause of severe acute kidney injury (AKI) in MM patients is tubulointerstitial nephropathy, which results from very high circulating concentrations of monoclonal immunoglobulin free light chains (FLC). In the setting of AKI, an early reduction of serum FLC concentration is related to kidney function recovery. The combination of extended high cutoff hemodialysis and chemotherapy results in sustained reductions in serum FLC concentration in the majority of patients and a high rate of independence from dialysis.

## 1. Introduction

Kidney dysfunction is a worldwide public health problem with an increasing incidence and prevalence, and it is associated with high costs and relatively poor outcomes [1]. Multiple myeloma (MM) is a clonal B-cell disease of proliferating plasma cells that mainly affects elderly and accounts for almost 10% of all hematologic malignancies [2]. High dose chemotherapy with autologous stem-cell transplantation (ACST) has become the standard strategy for newly young MM patients. However, the median duration of response after this procedure does not exceed 3 years, and few patients remain free of the disease for more than 10 years [3]. Relative survival rate is approximately 40% for 5 years and 20% for 10 years [4]. Kidney disease is a common and a potentially serious complication of MM that occurs in 20%–25% patients [5] and in up to 50% patients [6] during the course of their disease. It is possible to reverse kidney dysfunction in approximately 50% patients, but the remaining patients will have some degree of persistent chronic kidney disease (CKD); and of these, 2%–12% will require renal replacement therapy (RRT) [7]. Kidney dysfunction in MM may result from various factors, and in most cases it is minor and recovered easily with infusion solution and correction of serum calcium levels [5, 6], though occasionally the condition may become exacerbated. Both acute kidney injury (AKI) and progressive CKD can result in end-stage renal disease (ESRD). Persistent kidney dysfunction in MM is most commonly caused by tubular nephropathy due to monoclonal Ig secreted by the plasma cell clone, or a fragment thereof, most frequently a monoclonal light chain (LC) [8]. In this paper, we focus on the clinical management of the kidney dysfunction associated with MM.

## 2. Clinical Impact of Kidney Dysfunction in Multiple Myeloma

**Figure 1:**
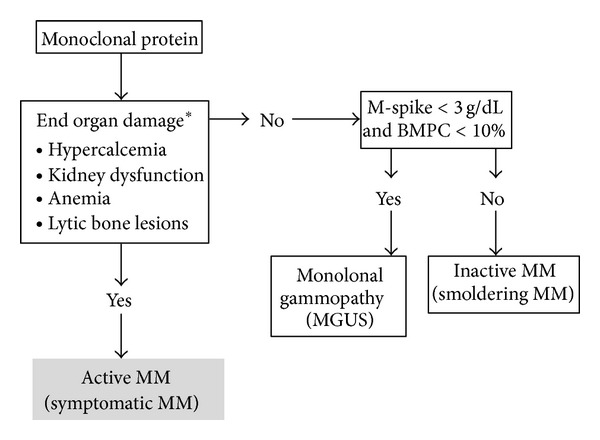
International Myeloma Working Group definition of multiple myeloma [9]. *MM-related organ damage includes the following: hypercalcemia [serum calcium > 0.25 mmol/L (1 mg/dL) above normal]; renal insufficiency (serum creatinine > 1.0 mg/dL above base line); anemia (hemoglobin > 2 g/dL below baseline); bone, lytic lesions, or osteoporosis with compression fracture; and symptomatic hyperviscosity, amyloidosis, or recurrent bacterial infections (>2 in 12 months). BMPC = bone marrow plasma cells.

Along with other clinical features including hypercalcemia, anemia, and lytic bone lesions, kidney dysfunction is a common complication in active MM (Figure 1) [9, 11]. Among newly diagnosed MM patients, 25%–50% present with kidney dysfunction, and approximately 9% require hemodialysis (HD) [5, 6]. Patients with AKI are more likely to experience early mortality and have worse overall survival [12, 13]. Before the introduction of the International Staging System (ISS) [14], the commonly used staging system for Durie and Salmon criteria [15], which was well known to be a good predictive indicator for prognosis in MM patients. Serum creatinine level was included in the staging system because it strongly predicted survival. However, as shown in Table 1, the estimated glomerular filtration rate (eGFR) was not accounted in ISS. In the 1980s, serum beta-2 microglobulin levels were identified as a strong prognostic factor in MM [15]. Recently, a risk score has been proposed that identified eGFR and beta-2 microglobulin levels as the capital predicting prognosis but did not include serum albumin levels because the unavailability of results for all patients [16]. The accumulation of the evidence suggests that kidney function is closely correlated with myeloma cell mass; that is, patients with a large tumor burden are more likely to have CKD. In the ISS cohort, 82% patients with levels ≥177 mmol/L were in stage III disease [14]. Cast nephropathy, also called myeloma kidney, is the most common cause of CKD, followed by amyloid light chain (AL)-type amyloidosis and monoclonal Ig deposition disease (MIDD) [17, 18].  Table 2 summarizes the association between clinical manifestations and various types of kidney injury in MM patients [10]. 

## 3. Acute Kidney Injury in Multiple Myeloma

**Figure 2:**
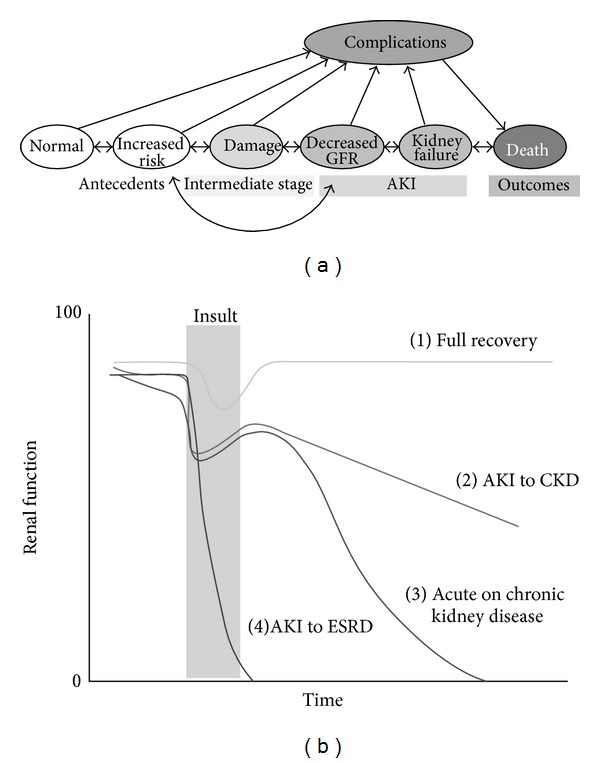
Acute kidney injury and progression to CKD [19]. (a) Conceptual model of acute kidney injury (AKI). (b) Natural history of AKI. Patients who develop AKI may experience (1) complete recovery of renal function, (2) development of progressive chronic kidney disease (CKD), (3) exacerbation of the rate of progression of preexisting CKD, or (4) irreversible loss of kidney function and evolve into ESRD.

AKI is defined as a sudden decrease in kidney function. AKI is one of the serious conditions that affect the structure and function of kidneys. It is a broad clinical syndrome, including specific diseases affecting the kidney such as MM. Even a minor acute reduction in kidney function correlates to an adverse prognosis. A schematic view of the conceivable course of AKI has been proposed (Figure 2) [19]. AKI could be an important cause of CKD or ESRD. Therefore, early detection and treatment of AKI would improve outcomes. Two criteria of AKI, which were based on sCr and urine output, the Risk, Injury, Failure, Loss, End-Stage Renal Disease (RIFLE) [21] and Acute Kidney Injury Network (AKIN) [22] have been proposed and validated. Recently, severity of AKI staged by RIFLE criteria (OR = 2.04 Failure stages versus Risk and Injury stage *P* = 0.06) has been reported as associated with marginally better long-term outcome in MM patients [23]. In 2012, the Kidney Disease: Improving Global Outcomes (KDIGO) AKI Guideline Work Group accepted the existing criteria for the diagnosis and staging of AKI and proposed a single definition of AKI that should be useful for practice, research, and public health (Table 3) [20]. It is widely accepted that GFR is the most useful kidney function index, and changes in sCr levels and urine output are surrogates marker for changes in GFR. In the clinical settings, an abrupt decline of GFR is detected as an increase in sCr levels. Although a small creatinine increase will predict adverse outcomes, the limitations of serum creatinine for early detection and accurate estimation of renal injury in AKI are well known [24]. Recently, AKI biomarkers have been developed to facilitate early detection, differential diagnosis, and prognosis. Among them, novel biomarkers such as urinary L-type fatty acid-binding protein (L-FABP) or neutrophil gelatinase-associated lipocalin (NGAL) are considered to reflect tubular epithelial cell injury [25, 26]. 

In patients with suspected MM, monoclonal heavy or light chains, known as Bence-Jones protein, should be analyzed in concentrated urine using electrophoresis with immunofixation of any identified protein bands in accordance with current myelomas guidelines [27]. Coincidence measurement of serum/urine albumin should be performed when the possibility of immunoglobulin light chain (AL) amyloid or monoclonal Ig deposition disease (MIDD) is suspected. The casts contain monoclonal free light chains (FLC) and Tamm-Horsfall glycoproteins and have been shown to acutely depress single nephron glomerular filtration rate [28]. The FLCs are freely filtered by the glomerulus and taken up by mesangial cells (toxicity to which may cause amyloidosis or light chain deposition disease) or tubular epithelial cells, where they can activate nuclear factor kappa beta (NF-kB) and cause apoptosis or epithelial-mesenchymal transition, leading to transcription of inflammatory cytokines. Recruitment of inflammatory cells to the interstitium ensues, promoting fibrosis [29]. 

Cast nephropathy is nearly always observed in advanced MM, when production of large amounts of LC overwhelms the capacity of catabolism in proximal tubules [8]. This nephropathy is usually triggered by several factors that increase urine FLC concentration. These factors include dehydration, hypercalcemia, infections, contrast medium usage, or use of nephrotoxic medications, including NSAIDs, diuretics, angiotensin-conversing enzyme inhibitors (ACEI), and angiotensin II receptor blockers (ARB). Also, patients with high serum monoclonal FLC (>500 mg/L) have a risk of developing AKI [30]. Even in the setting of severe kidney dysfunction, the serum FLC assay is a sensitive and specific screening tool [31]. The lack of sensitivity of serum protein electrophoresis in the detection of monoclonal FLC [32], which causes cast nephropathy, makes this test inappropriate as a screening tool, particularly in the setting of AKI. Because cast nephropathy develops in MM, the diagnosis may be straightforward but can become a challenge when the underlying myeloma has not yet been identified. 

## 4. Chronic Kidney Disease in Multiple Myeloma

**Figure 3:**
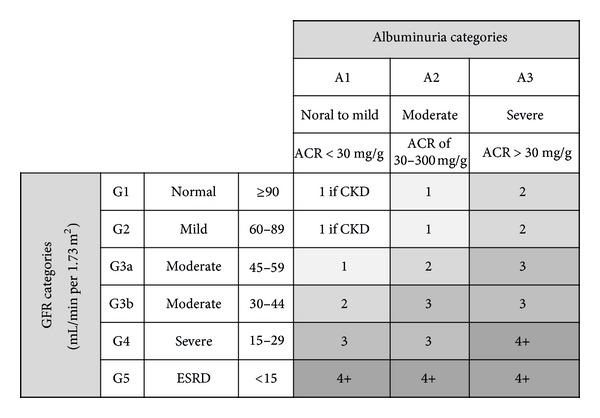
Guide to frequency of monitoring by GFR and albuminuria categories [33]. This GFR and albuminuria grid reflects the risk for progression by intensity. The numbers in the boxes are a guide to the frequency of monitoring (number of times per year). ACR = albumin – creatinine ratio; CKD = chronic kidney disease; GFR = glomerular filtration rate.

There is an even higher prevalence of the earlier stages of CKD, with adverse outcomes, including loss of kidney function, cardiovascular disease (CVD), and premature death. The KDIGO organization developed clinical practice guidelines in 2012 to provide guidance on the evaluation, management, and treatment of CKD (Table 4) [33]. Diagnostic thresholds of GFR of less than 60 mL/min/1.73 m^2^ and an albumin-creatinine ratio (ACR) of 30 mg/g or greater were retained. The exact frequency of GFR and ACR monitoring will depend on the severity of CKD Figure 3 [33] and the risk and rate of progression. The International Myeloma Working Group (IMWG) has recommended the use of the Modification of Diet in Renal Disease (MDRD) formula for the estimation of GFR in MM patients with stabilized sCr [34] as well as the KDIGO classification for the classification of CKD in MM [1, 35].

Factors associated with progression include cause of CKD, level of GFR, level of albuminuria, AKI, age, gender, race or ethnicity, elevated BP, hyperglycemia, dyslipidemia, smoking, obesity, history of cardiovascular disease, and ongoing exposure to nephrotoxic agents. The cause of CKD has been traditionally assigned based on presence or absence of underlying systemic diseases and location of known or presumed pathological abnormalities. The distinction between systemic diseases affecting the kidney and primary kidney diseases is based on the origin and locus of the disease process. In primary kidney disease the process arises and is confined to the kidney, whereas in systemic diseases the kidney is only one victim of a specific process, for example, diabetes. Certain genetic diseases cross this boundary by affecting different tissues, for example, adult polycystic kidney disease. The location of pathological and anatomical findings is based on the magnitude of proteinuria and findings from the urine sediment examination, imaging, and renal biopsy. In MM patients, CKD occurs mainly as a result of damage caused to renal tubules by FLCs (cast nephropathy). A variety of other nephrotoxic processes may also contribute to this damage including dehydration, hypercalcemia, nephrotoxic drugs, and infection.  Table 5 represents an example of a classification of causes of kidney diseases based on these two domains. MM is classified as tubulointerstitial disease in systemic disease affecting the kidney.

## 5. Kidney Dysfunction, and Chemotherapy, and Stem-Cell Transplant

In the era of conventional chemotherapy, several studies have confirmed that CKD is associated with poor prognosis in MM, with a median survival of <2 years [36–38]. Effective treatment of MM is the best management strategy for complicating kidney dysfunction. Melphalan-prednisone (MP) was established as the standard treatment in a trial involving 183 patients, which demonstrated that it prolonged the survival by 6 months compared with the use of melphalan alone [39]. Because melphalan is more likely to cause hematological toxicity in CKD patients, dose modification is needed [34]. Autologous stem cell transplantation (ASCT) with high-dose chemotherapy has been shown to improve the overall survival [40]. However, ASCT has been considered as an option for selected CKD patients because kidney dysfunction was associated with a shorter overall survival [41]. 

Since 2005, the treatment strategy for MM has significantly changed because of the successful introduction of new therapeutic agents. Three drugs, a proteasome inhibitor (bortezomib) and two immunomodulatory drugs (IMiDs, lenalidomide, and thalidomide), are referred to as novel agents, and each drug has a characteristic efficacy. Notably, there are reports of hyperkalemia occurring with the use of thalidomide in patients with severe CKD (including those on RRT); thus, at present, its usage requires caution [42, 43]. Thalidomide is metabolized by hydrolysis in serum and can be used without dose modification in severe CKD. Lenalidomide is a 4-amino substituted analog of thalidomide, which was first shown to be useful in the treatment of relapsed MM, though patients with advanced CKD were more likely to become thrombocytopenic or require dose reduction or interruption of lenalidomide [3, 44]. 

While these agents can be expected to restore kidney function by improvement in the primary disease, bortezomib, with a strong antitumor effect, is reported to rapidly improve kidney function [45]. Bortezomib was first administered to treat relapsed or refractory MM but has also shown to be effective as front-line therapy [46]. Bortezomib is cleared via hepatic oxidative deboronation [44], and so doses do not require adjustment in CKD [34]. The kidney response rate is based on improving creatinine clearance and response time, which were 59% and 1.8 months (traditional chemotherapy), 79% and 1.6 months (IMiDs), and 94% and 0.69 month (bortezomib), respectively [47]. The introduction of novel agents has led to an improved survival of patients with MM [48, 49], even in those with CKD.

## 6. Apheresis Therapy in Multiple Myeloma

There are several types of apheresis therapy that are applicable in MM patients. Plasma exchange (PE) or plasmapheresis involves the separation and removal of the blood cells and other substances from the plasma by centrifugation (based on cell density) or ultrafiltration using large-pore hemofilters (based on molecular size) [50]. This method is used to remove pathogenic substances, including autoreactive antibodies, immune complexes, paraproteins, lipoproteins, and inflammatory mediators such as cytokines. Fluid replacement after PE maintains normal plasma volume and electrolyte concentrations. Plasma filters have a pore size of approximately 0.3 *μ*m and membrane area of 0.1–0.8 m [2]. Homogenization of pore size has been sought to decrease cell leakage and hemolysis. The PE circuit includes the plasma filter, circuits for blood cells and plasma, equipment for plasma exchange (blood pump, plasma pump, hemadynamometer, plasma filtration manometer, trans-membrane-pressure (TMP) manometer, and an anticoagulant pump). A circuit for fluid replacement should be prepared when HD or hemodiafiltration is combined with PE. The ideal replacement solution should maintain normovolemia and normal plasma electrolyte concentrations. The choice of replacement fluid includes crystalloids, semisynthetic colloids (hetastarch, gelatin, and dextrans), human albumin solutions, liquid stored plasma, fresh-frozen plasma (FFP), and cryoprecipitate. The replacement solutions most commonly used are liquid stored plasma and human albumin solution for the removal of some pathogenic substances. FFP infusion can cause hypocalcemia as a result of calcium chelation by sodium citrate, and alkalosis and sodium overload can also occur. The hypotensive effects of citrate-induced hypocalcemia can be minimized by administering calcium gluconate as a continuous intravenous infusion and monitoring serum calcium levels. The treatment of choice for patients with AKI is combined plasmapheresis and HD to correct electrolyte abnormalities and provide renal support. A high flow volume may be needed when combining HD or hemodiafiltration with PE in patients undergoing long-term HD. When combining PE with HD in a serial circuit, a medical practitioner should monitor the procedure and stop it, if necessary, to prevent overfiltration at the HD side caused by decreased or obstructed blood flow at the PE side. Double-filtration plasmapheresis (DFPP) is a PE in which two filters with different pore sizes are used to separate toxic substances from plasma. The two-stage filtration allows the removal of albumin and its return into the blood circulation. This feature provides the advantage of decreasing the need for replacement fluid and its associated complications, including allergic reaction and infection, that can occur with PE. Using DFPP also decreases the high cost associated with the replacement fluid [51]. Cryofiltration is a modification of DFPP that involves cooling the separated plasma at the plasma separator (first membrane) to gelatinize the proteins in the plasma, which are then ablated at the large-pore plasma component separator (second membrane) [52]. The gelatinized and ablated proteins form cryoglobulin or cryogel. Cryoglobulin is the collective term for abnormal proteins, including single immunoglobulins and multiimmunoglobulins (mainly IgG or IgM) that clump into a gel at 39.2°F and dissolve at 98.6°F. Cryogel is a complex of heparin, fibronectin, and fibrinogen. Cryofiltration is used to treat patients with cryoglobulinemia, a medical condition in which the blood contains large amounts of cryoglobulins. Patients may have essential cryoglobulinemia or secondary cryoglobulinemia associated with various diseases, including macroglobulinemia, MM, connective tissue disease, and hepatitis C infection. 

FLC removal by apheresis therapies has been investigated as a means of preserving kidney function. The initial treatment investigated was PE, which has been tested in three trials [53–55], and overall there is no evidence of benefit. Two early trials had methodological fallacy. The first compared PE and HD with peritoneal dialysis (PD) alone [54]; the second was small; and the two groups were significantly different in terms of baseline prognostic factors. The lack of efficacy of PE would not be surprising. Light chains are so small (*κ*, 25 kDa; *λ*, 50 kDa) that they equilibrate between the intravascular and extra vascular compartments; thus, the intravascular compartment may only contain 20% of the total capacity. Namely, a standard series of single PE session might remove only 65% of intravascular FLCs [56]. However, rapid removal of FLC with PE in combination with chemotherapy could prevent further kidney dysfunction. A previous trial failed to show evidence that PE improved the outcome in patients with MM and AKI [57]. In this randomized controlled study of 107 patients who developed AKI after the diagnosis of MM, PE (5–7 exchanges of 50 mL/kg body weight) coupled with a chemotherapy regimen based on VAD (vincristine, doxorubicin, and dexamethasone) or MP (described previously) did not show significant effect on a composite criterion defined by death, RRT-dependent ESRD, or ESRD with a GFR <30 mL/min/1.73 m^2^, compared with chemotherapy alone. In this study, FLC levels were not measured, and histologic evidence of cast nephropathy was insufficient. Recently, Leung et al. have suggested that histological confirmation of cast nephropathy should be considered to analyze the effects of PE. In a retrospective series of 40 patients with MM-associated CKD, 18 cases had cast nephropathy that was biopsy proven. In their study including patients with cast nephropathy, the combination of PE with high-dose dexamethasone-based chemotherapy induced an attenuation of kidney dysfunction in 45% and in 75% of patients in whom serum FLC levels decreased by >50% with treatment. In contrast, no correlation between renal response and reduction in serum FLC levels was observed in another study including patients without biopsy-proven cast nephropathy [18], indicating that pathological confirmation might influence therapeutic strategy and prognosis in MM with CKD. A treatment strategy was recently designed combining bortezomib-based therapy and PE in patients with biopsy-proven cast nephropathy or a high probability of cast nephropathy (>200 mg/dL of FLC) [58]. The reported renal recovery rate of 86% will probably lead to improved patient survival. However, the relative contribution of PE prognosis improvement was not apparent. Therefore, these results should be interpreted with caution. The early decrease in FLC concentrations probably represents efficacy of the chemotherapy rather than that of PE [59, 60].

## 7. Dialysis Therapy in Multiple Myeloma

CKD is a common clinical feature of MM. Even with aggressive treatment, progression to ESRD occurs in up to 65% patients with cast nephropathy within 3 months of diagnosis [61]. Treatment-related mortality (29%) and morbidity (3.4%) are higher in patients with CKD than in patients with normal kidney function [15]. The unadjusted median overall survival (OS) on HD was 0.91 years in patients with MM and 4.46 years in non-MM patients [62]. With a review of the United States Renal Data System, MM-induced CKD is a considerable burden [63]. Of the 375 152 patients in the registry who initiated HD for ESRD, 3298 (0.88%) patients had MM. The 2-year all-cause mortality of patients with ESRD due to MM was 58% versus 31% in all other patients (*P* < 0.01) [63]. MM patients with progressive CKD have a tendency to die within 2–9 months after the diagnosis [64, 65]. If patients who die within 2 months of diagnosis are excluded, the median survival of patients with MM with ESRD is almost 2 years, and 30% survive for over 3 years [66, 67]. Similarly, another report showed that from 1985 to 2005, 1.5% (2453) of the 159 637 patients placed on RRT had MM [34]. The incidence of RRT for ESRD due to MM increased from 0.70 per million people (1986 to 1990) to 2.52 per million people (2001 to 2005) [34]. Some studies have also indicated that reversibility of kidney dysfunction is associated with improved survival [12, 13, 68]. Even patients who have not been diagnosed with MM at the time HD was initiated for ESRD are at risk of MM for several years, with odds ratios of 3.7, 1.9, 0.9, and 0.8 for 0–12 months, 12–25 months, 25–44 months, and >44 months after starting HD, respectively [69]. According to the recent report, between 0.9 and 1.5% of patients initiating maintenance HD suffer from MM, which may reflect therapeutic success because patients in whom renal function is not completely recovered survive long enough to be chronically dialyzed [62]. Patients with MM and ESRD can be treated either with HD or PD, and both seem to be equally effective [7, 70]. Patients who recover their renal function and obtain independence from HD have the same good prognosis as those who never developed AKI. Blade et al. reported that hypercalcemia, degree of renal failure, and amount of proteinuria are factors associated with renal dysfunction in MM-associated CKD patients [12]. We previously showed that degree of serum beta-2 microglobulin and hypercalcemia in MM-associated HD patients were significant and independent prognostic factors for predicting the probability of recovery from severe renal failure and discontinuation of HD [71]. Erythropoiesis-stimulating agents (ESAs) are agents similar to the cytokine erythropoietin, which stimulates red blood cell production (erythropoiesis). They can be used in patients with MM on HD to decrease transfusion requirements, although some studies suggest that they may decrease the overall survival [44, 72].

Besides the theoretical limitations of PE [58–60], high cutoff dialyzers have been verified in patients with myeloma kidney. These dialyzers have membranes with very large pores, allowing the passage of molecules up to 60–65 kDa, through which light chains can pass. An early analysis of this method suggested that up to 90% of light chains can be removed with 3 weeks of extended daily HD, while PE might remove only 25% of the total amount during the same period [73]. However, this success rate is dependent on the plasma cell clone responding to chemotherapy. The combination of extended high cutoff hemodialysis (HCO-HD) and chemotherapy was recently shown to result in sustained decrease in serum FLC concentrations in the majority of patients and a high rate of dialysis independence [74, 75].

## 8. Conclusion

Kidney dysfunction is a common feature of symptomatic MM and may cause major problems in clinical management. Its management remains challenging. Cast nephropathy is the most common cause of severe kidney dysfunction in MM. Serum FLC concentrations should be considered in MM patients with AKI. The successful introduction of new therapeutic agents and novel techniques for serum FLC removal has profoundly altered the therapeutic approach toward patients with cast nephropathy. Long-term dialysis is an efficacious treatment for patients with MM and ESRD. FLC removal with a combination of HCO-HD and chemotherapy may lead to early decrease in serum FLC concentrations and ameliorate AKI complicating MM.

## Figures and Tables

**Table 1:** The Durie-Salmon and International Staging systems criteria.

Stage	Durie-Salmon criteria	International Staging system criteria
I	All of the following: hemoglobin value > 10 g/dL, serum calcium value normal or ≤3 mmol/L bone X-ray, normal bone structure (scale 0), or solitary bone plasmacytoma only Low M-component production rate (IgG value < 5 g/dL; IgA value < 3 g/dL; Bence-Jones protein < 4 g/24 h)	Beta-2 microglobulin < 3.5 mmol/L and albumin ≥ 3.5 g/dL

II	Neither stage I nor stage III	Neither stage I nor stage III

III	One or more of the following: hemoglobin value < 8.5 g/dL serum calcium value > 3 mmol/L, advanced lytic bone lesions (scale 3) High M-component production rate (IgG value > 7 g/dL; IgA value > 5 g/dL; Bence-Jones protein > 12 g/24 h)	Beta-2 microglobulin > 5.5 mmol/L

Durie-Salmon subclassifications: relatively normal renal function (serum creatinine level < 177 mmol/L [<2 mg/dL]). Abnormal renal function (serum creatinine level ≥ 177 mmol/L [≥2 mg/dL]).

**Table 2:** Associations between clinical manifestations and types of kidney injury in MM [10].

Predominant renal syndrome	Major types of renal lesions
Acute kidney injury (AKI)	Myeloma cast nephropathy
	Acute tubular necrosis
	Iatrogenic effects
	Direct infiltration of renal parenchyma
	Acute tubulointerstitial nephropathy

Proteinuria/nephrotic syndrome	Monoclonal Ig deposition disease (MIDD)
	Amyloidosis
	Rare types of glomerular involvement

Chronic kidney disease (CKD)	Amyloidosis
	Myeloma cast nephropathy
	Monoclonal Ig deposition disease (MIDD)

Fanconi syndrome	Proximal tubulopathy

**Table 3:** Staging of acute kidney injury [20].

Stage	Serum creatinine	Urine output
1	1.5–1.9 times baseline OR ≥0.3 mg/dL (≥26.5 mmol/L) increase	<0.5 mL/kg/h for 6–12 h

2	2.0–2.9 times baseline	<0.5 mL/kg/h for ≥12 h

3	3.0 times baseline OR Increase in serum creatinine to ≥4.0 mg/dL (≥353.6 mmol/L) OR Initiation of renal replacement therapy OR, in patients <18 years, decrease in eGFR to <35 ml/min per 1.73 m^2^	<0.3 mL/kg/h for ≥24 h OR Anuria for ≥12 h

**Table 4:** Criteria for chronic kidney disease [33].

Markers of kidney damage (for >3 months)	
Albuminuria (AER ≥ 30 mg/dL; ACR ≥ 30 mg/g)	
Urinary sediment abnormalities	
Electrolyte and other abnormalities due to tubular disorders	
Abnormalities detected by histology	
Structural abnormalities detected by imaging	
History of kidney transplantation	
Decreased GFR (for >3 months)	
GFR < 60 mL/min per 1.73 m^2^ (GFR categories G3a–G5)	

ACR: albumin-creatinine ratio; AER: albumin excretion rate; GFR: glomerular filtration rate.

**Table 5:** Classification of CKD based on presence or absence of systemic disease and location within the kidney of pathologic-anatomic findings [33].

	Examples of systemic diseases affecting the kidney	Examples of primary kidney diseases
Glomerular diseases	Diabetes, systemic autoimmune diseases, systemic infections, drugs, and neoplasia (including amyloidosis)	Diffuse, focal, or crescentic proliferative GN, focal and segmental glomerulosclerosis, membranous nephropathy, and minimal change disease
Tubulointerstitial diseases	Systemic infections, autoimmune, sarcoidosis, drugs, urate, and environmental toxins, *Multiple myeloma *	Urinary-tract infections, stones, and obstruction
Vascular diseases	Atherosclerosis, hypertension, ischemia, cholesterol emboli, systemic vasculitis, thrombotic microangiopathy, and systemic sclerosis	ANCA-associated renal limited vasculitis and fibromuscular dysplasia
Cystic and congenital diseases	Polycystic kidney disease, Alport syndrome, and Fabry disease	Renal dysplasia, medullary cystic disease, and podocytopathies
